# The *Smn*-Independent Beneficial Effects of Trichostatin A on an Intermediate Mouse Model of Spinal Muscular Atrophy

**DOI:** 10.1371/journal.pone.0101225

**Published:** 2014-07-01

**Authors:** Hong Liu, Armin Yazdani, Lyndsay M. Murray, Ariane Beauvais, Rashmi Kothary

**Affiliations:** 1 Regenerative Medicine Program, Ottawa Hospital Research Institute, Ottawa, Ontario, Canada; 2 Department of Cellular and Molecular Medicine, University of Ottawa, Ottawa, Ontario, Canada; 3 Department of Medicine, University of Ottawa, Ottawa, Ontario, Canada; University of Montréal and Hôpital Maisonneuve-Rosemont, Canada

## Abstract

Spinal muscular atrophy is an autosomal recessive neuromuscular disease characterized by the progressive loss of alpha motor neurons in the spinal cord. Trichostatin A (TSA) is a histone deacetylase inhibitor with beneficial effects in spinal muscular atrophy mouse models that carry the human *SMN2* transgene. It is currently unclear whether TSA specifically targets the *SMN2* gene or whether other genes respond to TSA and in turn provide neuroprotection in SMA mice. We have taken advantage of the *Smn^2B/-^* mouse model that does not harbor the human *SMN2* transgene, to test the hypothesis that TSA has its beneficial effects through a non-SMN mediated pathway. TSA increased the median lifespan of *Smn^2B/-^* mice from twenty days to eight weeks. As well, there was a significant attenuation of weight loss and improved motor behavior. Pen test and righting reflex both showed significant improvement, and motor neurons in the spinal cord of *Smn^2B/-^* mice were protected from degeneration. Both the size and maturity of neuromuscular junctions were significantly improved in TSA treated *Smn^2B/-^* mice. Of interest, TSA treatment did not increase the levels of Smn protein in mouse embryonic fibroblasts or myoblasts obtained from the *Smn^2B/-^* mice. In addition, no change in the level of *Smn* transcripts or protein in the brain or spinal cord of TSA-treated SMA model mice was observed. Furthermore, TSA did not increase Smn protein levels in the hind limb muscle, heart, or liver of *Smn^2B/-^* mice. We therefore conclude that TSA likely exerts its effects independent of the endogenous mouse *Smn* gene. As such, identification of the pathways regulated by TSA in the *Smn^2B/-^* mice could lead to the development of novel therapeutics for treating SMA.

## Introduction

Spinal Muscular Atrophy (SMA) is an autosomal recessive neuromuscular disorder characterized by the progressive loss of alpha motor neurons in the anterior horn region of the spinal cord. SMA is caused by homozygous deletions or rare missense mutations in the survival motor neuron 1 (*SMN1*) gene [1]. Although SMA is caused by deletions in *SMN1*, all patients retain at least one copy of the near identical *SMN2* gene. *SMN1* produces full-length *SMN* transcript and protein while *SMN2* produces only 10% full-length *SMN* transcript and protein, and therefore 90% of *SMN2* transcript lacks exon 7 and encodes an unstable truncated protein [2], [3]. The severity of the disease in SMA is inversely correlated with *SMN2* gene copy number [4], [5]. Unlike humans, rodent species only carry a single survival of motor neuron gene (*Smn*) and its inactivation leads to early embryonic lethality [6]. A number of strategies have been employed to generate viable mouse models of SMA. Some of the most commonly used models rely on the addition of a human *SMN2* transgene onto an *Smn* ^-/-^ background [7], [8]. For example, the *Smn^-/-^;SMN2;SMNΔ7* mouse model of SMA (herein referred to as the delta 7 mouse model) contains 2 copies of the human *SMN2* onto a *Smn* ^-/-^ background and also carries 2 copies of the delta 7 *SMN* cDNA. An alternative approach has been to introduce a three-nucleotide substitution in the exon 7 splicing enhancer of the murine *Smn* gene, generating a model known as the *Smn^2B/-^* mouse model [9], [10].

Numerous strategies have been proposed for treating SMA, including the use of histone deacetylase (HDAC) inhibitors. HDAC inhibitors can modify the chromatin architecture by relaxing the chromatin conformation through a net increase in the acetylation state of histones. HDAC inhibitors have been shown to increase *SMN2* expression through direct modification of histone acetylation state at the *SMN2* promoter [11]. Indeed, a number of different HDAC inhibitors have had promising results in mouse models of SMA that carry the *SMN2* transgene [12], [13], [14], [15], [16]. Specifically, trichostatin A (TSA) has produced some of the most dramatic results, increasing life span of the delta 7 mouse model by 19% [13]. However the exact mechanism for the beneficial effects of HDAC inhibitors in SMA are currently unclear. These beneficial effects may be mediated via the small but significant increase in SMN levels observed. Numerous studies have now demonstrated that very small increases in SMN protein level can have a dramatic effect upon the phenotype of mouse models. They further demonstrated that TSA can act directly upon the SMN2 transgene to increase its' expression [11]. However, it should also be noted that HDAC inhibitors can act globally across the genome and orchestrate the activity of many genes to provide neuroprotection. For this reason, a number of HDAC inhibitors have been used in clinical trials for a variety of other neurodegenerative conditions. Therefore the identification of novel pathways that TSA may regulate for neuroprotection could provide new therapeutic avenues in treating SMA.

In this study, we assessed whether TSA can have beneficial effects on an SMA mouse model in the absence of the *SMN2* transgene. We have taken advantage of the *Smn^2B/-^*intermediate mouse model of SMA, which does not carry human *SMN2*. Here we show that TSA treatment significantly improved survival, reduced weight loss and improved motor behavior in *Smn^2B/-^* mice. Daily administration of TSA reduced motor neuron loss in the spinal cord, improved the maturity and size of the neuromuscular junctions (NMJs), and improved myofiber histology in the tibialis anterior (TA) muscle. Importantly, administration of TSA led to no increase in Smn transcript or protein levels in brain, spinal cord, muscle or liver of *Smn^2B/-^* mice. We also observed no increase in Smn protein levels in TSA treated embryonic fibroblasts and myoblasts cultured from *Smn^2B/-^* mice. We therefore suggest that the beneficial effects observed in this study are likely mediated independent of *Smn*.

## Materials and Methods

### Animals, intraperitoneal injection, growth and motor function test

The intermediate SMA mouse model, *Smn^2B/-^*, were established in our laboratory and maintained on a C57BL/6 x CD1 hybrid background [9], [10]. The *Smn^2B/+^* heterozygous littermates do not display any detectable phenotype and were used as littermate controls. TSA was dissolved in DMSO at a concentration of 4 µg/µl as described previously [13]. Mice were administered with TSA (10 mg/kg of body weight) or vehicle DMSO through intraperitoneal injection daily from postnatal day (P)12 to P25. Soft bedding, water-soaked food cubes and transgel were provided to the pups in TSA- and vehicle-treated groups after weaning at P21. Warm pads were also provided to *Smn^2B/-^* pups under the cages from P21 to P25. In order to eliminate the synergistic effects of diet and TSA [16], mice received a regular diet without any caloric or dietary enhancement. During TSA or vehicle treatment at P12-P25, mice were kept in the surgical surveillance area. Mice were monitored daily by animal facility professionals, and additionally monitored by the researchers once per day. Animal facility reported to researchers for any identified animal deaths and recommended to sacrifice animals in need. After the treatment period, mice were kept in the standard area. Animal facility professionals provided feeding and general monitoring daily and cage changing weekly. In addition, from P26 to P41, mice were monitored daily by the researchers. From P42 to P119, mice were monitored weekly by the researchers. The University of Ottawa Animal Care Committee approved all experimental protocols. The protocols conformed to or exceeded those defined in the Canadian Council on Animal Care's Guide to the Care and Use of Experimental Animals, and the Animals for Research Act.

The mice were weighed daily from P12 to P25. During the survival study, humane endpoints were applied as per veterinary recommendation (severe dehydration, low body temperature, and desertion by feeding mother). At endpoints, mice were anesthetised via intraperitoneal injection of tribromoethanol (Avertin) followed by instant cervical dislocation. Righting reflex test and pen test examined the mice for motor function. For righting reflex test, the mice were placed on their backs, and the time the mice took to right themselves was recorded. Thirty seconds was also defined as the maximum time. TSA and vehicle treated *Smn^2B/-^* mice were assessed daily from P12-P18. For pen test, the mice were placed on a pen and the time that a mouse took before it fell off was recorded. Average of three consistent records for each mouse was used for statistical analysis. Thirty seconds was set as the maximum testing time. TSA and vehicle treated *Smn^2B/-^* mice were assessed daily from P18 to P25.

### Histology, NMJ analysis and immunohistochemistry

To harvest tissues for histological examination, mice were transcardially perfused with 4% paraformaldehyde. Spinal cords from the L1 level downwards and tibialis anterior (TA) muscles at the thickest point were dissected and processed for paraffin embedding. Transverse sections of L1 spinal cord spanning 500 µm with intervals of 40 µm were processed for hematoxylin/eosin staining and motor neuron analysis. Three to five animals were analyzed for each treatment group. For each animal, six sections were examined. Images were taken on a Zeiss/Axioplan2 microscope with a Plan-Neofluar 20x/0.5 lens and processed using Adobe Photoshop CS software. Analyses were performed using the AxioVision Rel 4.6 program. Motor neuron cell bodies were determined by their morphology and a diameter of 18 µm or larger. Number of motor neuron cell bodies located at each side of the ventral horn below the horizontal line at the level of the central canal was recorded. The transversus abdominis (TVA) and rectus abdominis (RA) muscles were harvested and NMJs were labeled by immunohistochemistry as described previously [17], [18]. Briefly, muscles were immediately dissected from recently sacrificed mice and fixed in 4% paraformaldehyde (Electron Microscopy Science) in PBS for 15 min. Post-synaptic AChRs were labeled with alpha-bungarotoxin (aBTX) for 30 min. Muscles were permeabilized in 2% Triton X-100 in PBS for 30 min, then blocked in 4% bovine serum albumin (BSA)/1% Triton X-100 in PBS for 30 min before incubation overnight in primary antibodies [neurofilament (NF; 2H3) - Developmental Studies Hybridoma Bank; synaptic vesicle protein 2 (SV2) - Developmental Studies Hybridoma Bank] and visualized with cy3-conjugated secondary antibodies [Cy3 goat anti-mouse; all 1∶250, Jackson]. Muscles were then whole-mounted in Dako Fluorescent mounting media. Images were taken with a Zeiss LSM-510 meta confocal microscope. Histological analysis of the TA muscle was performed for assessing myofiber morphology.

### Cell culture and drug treatment

*Smn^2B/-^* mouse primary myoblasts were isolated from hind limb muscles of 3-week-old mice as originally described [19]. The primary myoblasts were cultured on collagen-coated plates (Gibco) and maintained under growth conditions using Hams F10 (Wisent) media supplemented with 20% fetal bovine serum (FBS), 2.5 ng/mL human recombinant basic fibroblast factor (Invitrogen), and 2% penicillin/streptomycin (Gibco). In all experiments, cells were maintained at 37°C with 5% CO_2_ and were provided fresh media every second day. The cells were grown and treated with 100 nM of TSA or DMSO immediately added to the media prior to each use for a 24 hr period and were maintained at 37°C with 5% CO_2_.

For nuclear extraction, the cells were harvested after 2 washes with PBS and trypsinized and pelleted followed by an additional wash in PBS. The cells were pelleted and resuspended (2–10*10^6^ cells/mL) in hypotonic buffer (10 mM Hepes pH 7.9, 1.5 mM MgCl_2_, 10 mM KCl, 0.5 µL 1 M DTT/mL, 1 µL 200 mM PMSF/mL). After resuspension, the cells were placed on ice for 5 minutes and then centrifuged at 13,000 rpm for 15 sec at 4°C. Hypotonic buffer was added at half the volume with 0.15% NP-40 and the cells were placed on ice for 5 minutes. The cells were then centrifuged at 13,000 rpm for 15 sec at 4°C and the nuclei were suspended in 0.2 N HCl and placed on a rotator at 4°C overnight. The cells were then centrifuged at 13,000 rpm for 15 minutes at 4°C and the supernatant that contains the histones was collected.

For the total protein isolation, the cells were treated with a single dose of TSA (100 nM) for a 24 hr period and were maintained at 37°C with 5% CO_2_. The cells were then washed twice with ice cold PBS and lysed using RIPA buffer. After the lysis on ice for 10 minutes, the cells were centrifuged at 13,000 rpm for 15 minutes and the supernatant collected.

*Smn^2B/-^* primary mouse embryonic fibroblasts (MEFs) were established from E13 embryos obtained from pregnant female mice. The uterine horns from the mice were dissected and rinsed with 70% ethanol and placed in PBS. Each embryo was carefully separated from the placenta and surrounding tissue and the brain and dark red organs were dissected out. The dissected head was used for genotyping performed by PCR. The embryo carcass was then washed with fresh PBS and blood removed. The embryo was then finely minced in PBS using razor blades. After this step, the obtained tissue was suspended in 2 mL of ice cold trypsin-EDTA and using gentle shaking was incubated at 37°C for 15 minutes. After sedimentation, the suspension was transferred to a 50 mL Falcon tube and mixed with 2 volumes of fresh MEF media which contained high glucose DMEM supplemented with 10% FBS, 1% L-glutamine, 1% penicillin/streptomycin. After incubation, the supernatant was removed and was subjected to low speed centrifugation for 5 minutes and the resulting pellet was resuspended in fresh warm MEF media and cells plated in a 10 cm dish. Each embryo was plated into a separate 10 cm dish and was maintained at 37°C with 5% CO_2_ until cells reached confluency (1–2 days). The experiments were performed at the 4^th^ passage and confluent plates were treated with TSA (100 nM) or DMSO for a 24 hr period. After treatment, the cells were lysed and total and/or nuclear protein was obtained.

For nuclear protein isolation, the cells were harvested after 2 washes with PBS and trypsinized and pelleted. The pellet was then washed with 10 mL of PBS and centrifuged at 300 g for 10 minutes. The pellet was then resuspended in 1 mL of hypotonic lysis buffer containing 10 mM Tris-HCl pH 8.0, 1 mM KCL, 1.5 mM MgCl_2_ and 1 mM DTT and incubated for 30 minutes on a rotator at 4°C. The intact nuclei preparation was centrifuged at 10,000 g for 10 minutes at 4°C. The pellet was then resuspended in 0.5 mL of 0.2 N HCl and incubated overnight on a rotator. The samples were then centrifuged at 16,000 g for 10 minutes and the supernatant was obtained for quantification.

### Reverse transcriptase-polymerase chain reaction (RT-PCR) and quantitative RT-PCR (qRT-PCR)

To harvest tissues for mRNA analysis, mice were sacrificed by cervical dislocation four hours after the last injection. Brains and spinal cords were dissected, frozen in liquid nitrogen, and stored at -80°C. RNA was extracted and RT-PCR performed as described previously [20] with the following primers: *Smn* long (194-1117): Forward 5′ GCA CAG CCA GAA GAA AAC CT 3′, Reverse 5′ CGA CAC GCA CAC TCC ACT 3′; *Follistatin*: Forward 5′ CCT TTC AAG TGG ATG ATT TTC 3′, Reverse 5′ ACA GTA GGC ATT ATT GGT CTG 3′; *β-actin*: Forward 5′ CCG TCA GGC AGC TCA TAG CTC TTC 3′, Reverse 5′ CTG AAC CCT AAG GCC AAC CGT 3′. For qRT-PCR, 1 µL of cDNA was used in a total reaction volume of 20 µL Supermix (BIORAD) according to the manufacturer's instructions. The PCR reaction was performed on a CFX connect (BIORAD) qPCR system with 40 amplification cycles. Changes in gene expression after TSA treatment were quantified based on the 2ΔCtDMSO-ΔCtTSA value. Ct was normalized to *Gapdh* (ΔCt = Ct target gene-Ct Gapdh). Triplicate samples were used for each PCR run. Primers for qPCR include: *Smn* (final concentration at 50 nM): Forward 5′ GCC ACA ACT CCC TTG AAA CAG 3′, Reverse 5′ TCG GGG AAA GTA GGT CAG ATA AG 3′; *Gapdh* (50 nM): Forward 5′ TGA AGG GGT CGT TGA TGG 3′, Reverse 5′ AAA ATG GTG AAG GTC GGT GT 3′.

### Histone isolation and western blot analysis

Histone isolation was performed using the protocol adapted from Mishra et al. [21]. One hundred mg of brain or liver tissue was homogenized using PowerGen tissue homogenizer (Fisher Scientific) in 500 µL of ice-cold histone lysis buffer (10 mM Tris-HCl, pH 6.5, 50 mM sodium bisulfite, 1% Triton X-100, 10 mM MgCl_2_, and 8.6% sucrose). For nuclear fraction isolation, the samples were centrifuged at low speed (1,000 g) for 10 minutes. This was followed by two additional washes in cold lysis buffer and once with cold Tris-EDTA (10 mM Tris-HCl, pH 7.4, and 13 mM EDTA). The nuclei were then resuspended in 100 µL of cold water followed by acidification with HCl at a final concentration of 0.2 N. The samples were vortexed and placed on a rotator for 1 hour. Acid soluble proteins were obtained using centrifugation at 15,000 g for 10 minutes and were precipitated in 1 mL acetone overnight at 25°C. Following this step, acetone precipitated proteins were collected by centrifugation at 15,000 g for 10 minutes and the protein pellets were air dried and suspended in 50 µL water. The Bradford assay was used for determination of protein concentration. SDS-PAGE and western blot were performed and membranes probed with a rabbit anti-acetylated H3 histone antibody (Millipore; 1∶5000), rabbit anti-acetylated H4 histone antibody (Millipore; 1∶5000), and anti-pan H3 histone antibody (Millipore; 1∶2000). The bands were visualized with a peroxidase-linked goat anti-rabbit IgG secondary antibody (Chemicon; diluted 1∶10,000) by chemiluminescence detection (Thermo Scientific Pierce ECL Western Blotting Substrate). Densitometry was performed using Image J software and acetylated histone H3 were normalized to pan-histone H3 values. To harvest tissues for total protein analysis, mice were sacrificed by cervical dislocation four hours after the last injection. Brains, spinal cords and livers were dissected, frozen in liquid nitrogen and stored at −80°C. Protein extracts were prepared, and SDS-PAGE and western blot performed as previously described [20]. The primary antibodies consisted of mouse monoclonal anti-Smn (BD Transduction Laboratory; 1∶5000), mouse monoclonal anti-GAPDH (Abcam; 1∶5000), and anti-β-tubulin (Developmental Studies Hybridoma Bank; clone E7; 1∶500). AlphaEaseFC software was used for quantification analysis.

### Statistical analysis

Statistical analyses were performed with Microsoft Office Excel and GraphPad Prism software (San Diego, CA). Analysis for statistical significance between groups was performed in Microsoft Excel. The dataset for each genotype and treatment group was compared to achieve the P value by Student's t-test (two-sample assuming equal variances and two-tailed). Y-axis values in the graphs were expressed as mean +/- standard deviation (SD) or standard error of the mean (SEM). P<0.05 was considered to indicate statistical significance.

## Results

### TSA treatment improves survival and motor function in *Smn^2B/-^* mice

**Figure 1:**
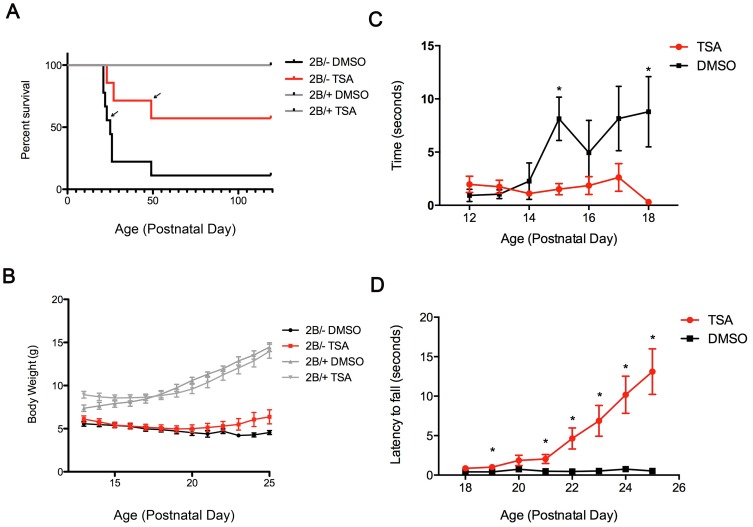
TSA treatment promotes survival, attenuates weight loss, and enhances motor function in *Smn^2B/-^* mice. (**A**) Kaplan-Meier Survival curves of TSA or vehicle (DMSO) treated *Smn^2B/-^* mice and littermate controls. Median lifespan is 8 weeks in TSA treated *Smn^2B/-^* mice (black arrow) in comparison to 23 days in DMSO-treated (black arrow). n = 9 (*Smn^2B/-^* DMSO), 7 (*Smn^2B/-^* TSA), 5 (*Smn^2B/+^* DMSO), 4 (*Smn^2B/+^* TSA). (**B**) Growth curves of TSA or vehicle (DMSO) treated *Smn^2B/-^* mice and littermate controls from P13 to P25. DMSO-treated *Smn^2B/-^* mice gradually lost weight during this time period. n = 16 (*Smn^2B/-^* DMSO), 15 (*Smn^2B/-^* TSA), 11(*Smn^2B/+^* DMSO), 14 (*Smn^2B/+^* TSA). (**C**) Righting reflex test. Mild improvement observed in the righting ability of TSA-treated *Smn^2B/-^* mice from P15 onwards. n = 16 (*Smn^2B/-^* DMSO) and 15 (*Smn^2B/-^* TSA). (**D**) Pen test assay on *Smn^2B/-^* mice treated with TSA or DMSO. Thirty seconds was set as the maximum time period. Improvement in latency to fall in TSA treated SMA mice (red line) became more apparent after P21 in comparison to DMSO treated SMA mice (black line). n = 16 (*Smn^2B/-^* DMSO) and 15 (*Smn^2B/-^* TSA).

Previous work has shown that TSA leads to improvements in survival and weight in the delta 7 mouse model of SMA [13]. Here, we tested the efficacy of TSA in the *Smn^2B/-^* mouse model, which does not carry the human *SMN2* transgene. TSA was delivered using daily intraperitoneal injections at a dose of 10 mg/kg from P12 to P25. Vehicle (DMSO) was used as a control. Daily administration of TSA led to a significant improvement in survival of *Smn^2B/-^* mice. Median lifespan of the *Smn^2B/-^* mice was increased from twenty days to eight weeks after TSA treatment (Fig. 1A). We also observed a trend towards improvements in growth of TSA treated *Smn^2B/-^* mice, however this did not reach statistical significance (Fig. 1B).

To investigate motor function, *Smn^2B/-^* mice were monitored daily to record righting reflex time from P12 to P18, and latency to fall using the pen test from P18 to P25. The TSA treated animals displayed faster righting times at P15 and P18 (Fig. 1C), and increased grip strength measured using the pen test from P21 to P25 (Fig. 1D).

Therefore, TSA treatment leads to significant improvement in survival and motor function. It is important to note that even after TSA treatment was discontinued at P25, the animals continued to survive and show signs of improvement past the normal lifespan of non-treated *Smn^2B/-^* mice. This provides further evidence that there is a critical time window in SMA pathogenesis.

### TSA improves the neuromuscular pathology that is observed in *Smn^2B/-^* mice

**Figure 2:**
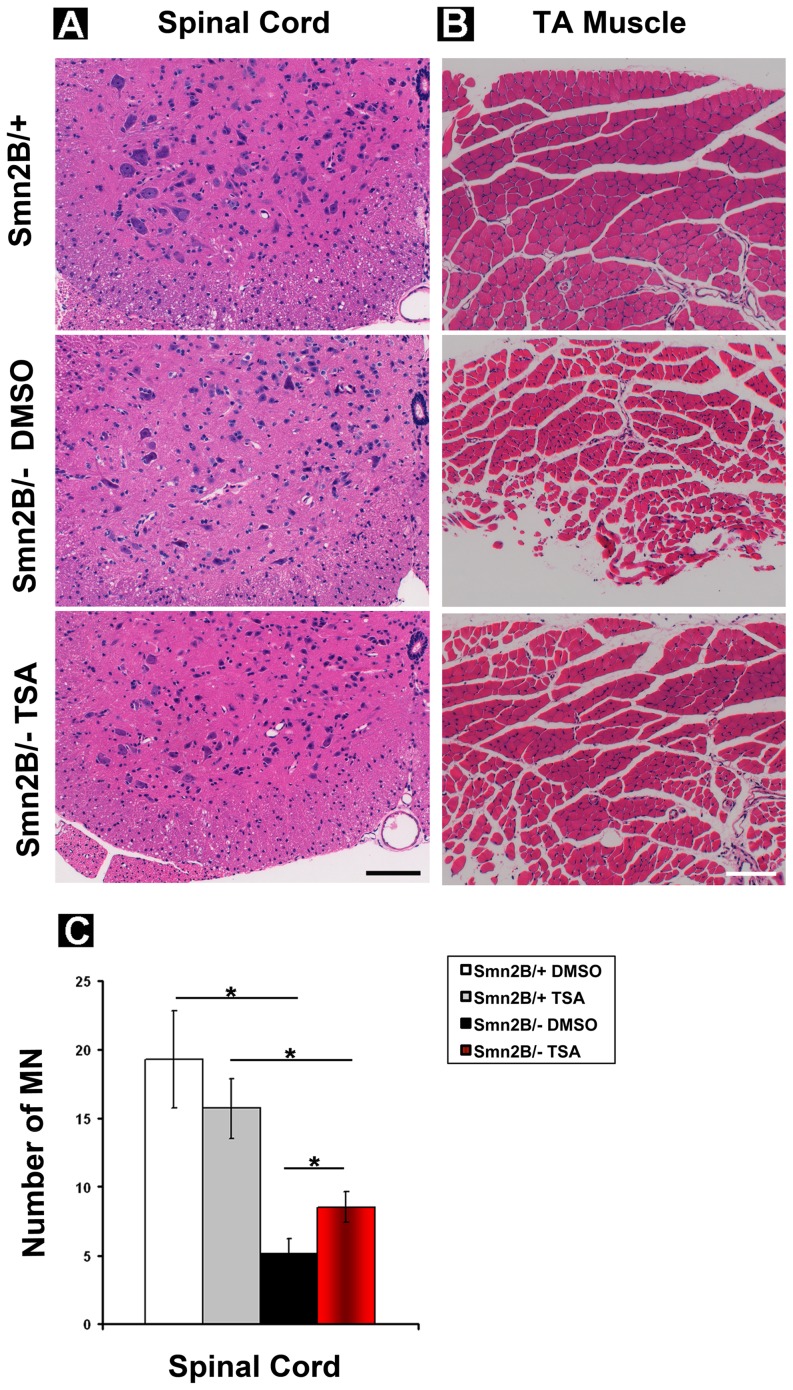
TSA treatment reduces motor neuron loss in the spinal cord and improves myofiber histology in *Smn^2B/-^* mice. (**A**) Cross-sections through the L1 lumbar spinal cord from *Smn^2B/+^* heterozygous control mice, and *Smn^2B/-^* mice treated with vehicle (DMSO) or TSA were processed for haematoxylin/eosin staining. Scale bar = 200 µm. (**B**) Cross-sections through the tibialis anterior (TA) muscles from *Smn^2B/+^* heterozygous control mice, and *Smn^2B/-^* mice treated with DMSO or TSA were processed for haematoxylin/eosin staining. Scale bar = 200 µm. (**C**) Quantification of motor neuron cell body number in the spinal cord from TSA or vehicle treated mice. TSA treatment results in a significant increase in the number of motor neurons in the spinal cord of *Smn^2B/-^* mice compared with DMSO treated *Smn^2B/-^* mice. n = 3 (*Smn^2B/-^* DMSO) and 4 (*Smn^2B/-^* TSA).

To investigate the cellular mechanisms underlying the phenotypic improvements observed in TSA treated mice, we investigated the effects of TSA on the neuromuscular pathology of *Smn^2B/-^* mice. Motor neuron loss in the anterior horn region of spinal cords is a key hallmark of SMA in both humans and *Smn^2B/-^* mice [9], [22]. Quantification of motor neuron cell body number revealed a statistically significant increase in TSA treated *Smn^2B/-^* mice compared with the DMSO treated controls (Fig. 2A and C). Interestingly, this data contrasts with previous work reporting that TSA was unable to reduce the motor neuron loss that is normally observed in the delta 7 model of SMA [13]. This is perhaps reflective of the relative difference in severity of these two SMA mouse models, with the more prolonged pathology being observed in the *Smn^2B/-^* mice.

**Figure 3:**
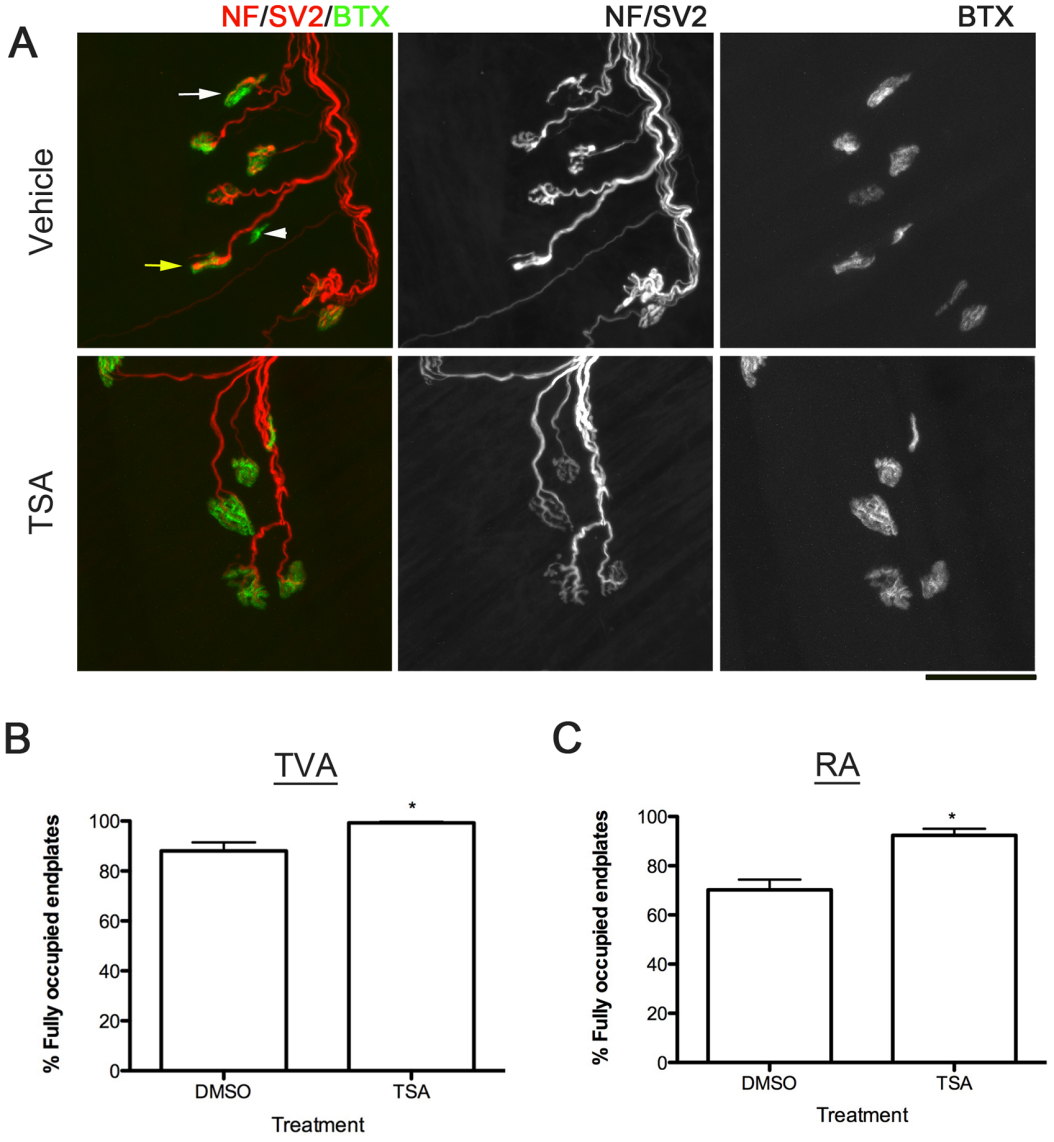
TSA administration decreases neuromuscular junction loss in *Smn^2B/-^* mice. (**A**) Confocal micrographs show neuromuscular junctions (NMJs) labelled with antibodies against neurofilament (NF, red), synaptic vesicle protein 2 (SV2, red) and alpha-bungarotoxin (BTX, green). Note that whilst there is widespread NMJ pathology in vehicle treated mice, as evidence by full/partial denervation (white arrowhead/arrow respectively), NF accumulation (yellow arrow) and small, immature post-synaptic endplates, NMJs appear much more healthy in TSA treated mice, as evidenced by a decrease in denervation and NF accumulation, and an increase in post-synaptic size and maturity (scale bar = 50 µm). (**B, C**) Bar charts show an increase in the percentage of fully occupied endplates in both the transversus abdominis (TVA; **B**) and rectus abdominis (RA; **C**) muscles following TSA treatment. * P<0.05 by Mann Whitney test.

We went on further to investigate whether the neuroprotective properties of TSA extends to the NMJ. *Smn^2B/-^* mice display significant levels of denervation, which is accompanied by a decrease in NMJ size and complexity [9]. This finding was recapitulated in our DMSO treated *Smn^2B/-^* mice, where we observed widespread NMJ pathology as evidenced by denervation, neurofilament accumulation and immature post-synaptic endplates (Fig. 3A, top panels). TSA administration significantly improved the overall appearance of *Smn^2B/-^* NMJs, with a decrease in neurofilament accumulation and an increase in post-synaptic size and maturity (Fig. 3A, bottom panels). Importantly, quantification of the percentage of fully occupied endplates also revealed a significant increase in TSA treated *Smn^2B/-^* mice compared to vehicle treated controls (Fig. 3B and C).

The data above demonstrate that TSA has neuroprotective properties at the level of motor neurons and NMJs. Since the neuromuscular pathology is significantly improved in *Smn^2B/-^* mice and this is accompanied by an improvement in motor behaviour, we investigated whether the beneficial effects could reach the muscle. *Smn^2B/-^* mice have smaller and more immature myofibers and display a delay in muscle development [23], [24], [25]. TSA treatment led to improvements in myofiber histology (Fig. 2B). We therefore conclude that TSA significantly improves the pervasive neuromuscular pathology that is evident in *Smn^2B/-^* mice; this consequently leads to positive functional outcomes such as improved survival, growth, and motor behavior.

### TSA administration does not result in an increase in *Smn* in *Smn^2B/-^* mice or cells

**Figure 4:**
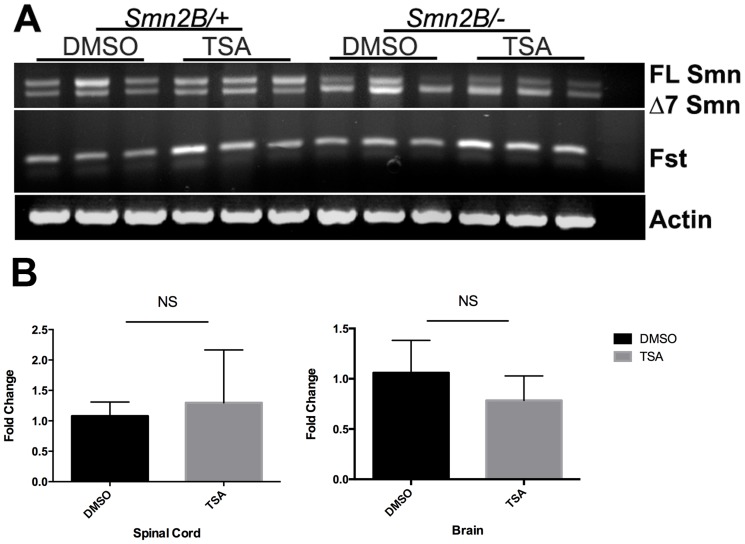
No alteration in the splicing pattern or levels of Smn mRNA in the brains of TSA-treated mice. (**A**) RT-PCR for *Smn* and a known target of TSA, *follistatin*, was conducted in total RNA extracted from brains of *Smn^2B/-^* mice or littermate controls treated with TSA or DMSO vehicle. Ratio of full length *Smn* versus Δ7*Smn* transcripts remained similar after TSA treatment in *Smn^2B/-^* or in control mice. Transcript level of *follistatin* is elevated in TSA-treated mice. Actin serves as a loading control. (**B**) Real-time quantitative RT-PCR analyses of RNA from spinal cord and brain. No significant change in the level of *Smn* transcripts (full length *Smn* plus Δ7*Smn*) was revealed after TSA treatment (n = 3). Fold increase normalized to reference gene *Gapdh*.

In previous clinical trials and studies of HDAC inhibitors in SMA patients and animal models, the beneficial effects have been attributed to the increase in SMN transcript and protein levels [13], [26], [15], [27]. We therefore investigated the effects of TSA on *Smn* expression in various tissues and primary cells obtained from the *Smn^2B/-^* mice. RT-PCR analysis of RNA from the spinal cord of *Smn^2B/-^* mice revealed that there is no change in the ratio of full-length and delta7 *Smn* splice isoforms following TSA treatment (Fig. 4A). Furthermore, TSA administration did not increase *Smn* transcript levels in the spinal cord or the brain of *Smn^2B/-^* mice (Fig. 4B). However, it is noteworthy that *follistatin* from the same animals was upregulated after treatment as a proof of TSA efficacy (Fig. 4A).

**Figure 5:**
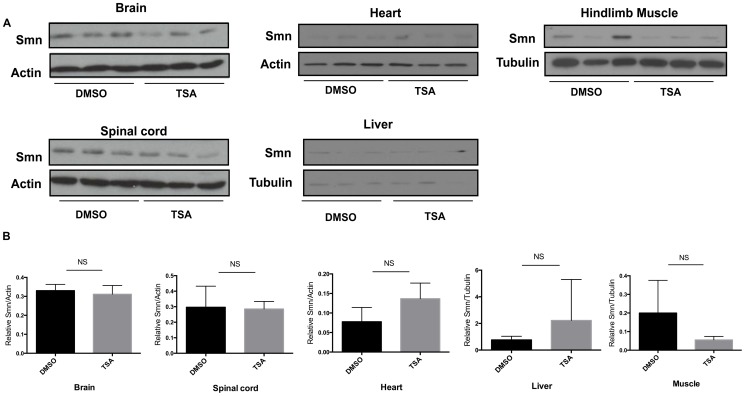
TSA treatment does not increase Smn protein levels. (**A**) Western blot analyses were performed with specific antibodies against Smn in protein extracts from brains, spinal cords, hind limb muscle, hearts, or livers of *Smn^2B/-^* mice treated with either TSA or DMSO vehicle. Each lane represents one animal. β-tubulin or actin serve as a loading controls. (**B**) Quantification revealed no significant increase of Smn protein levels in brains, spinal cords, hind limb muscle, hearts, or livers of TSA-treated SMA mice (n = 3).

Although there was no change in *Smn* transcript levels, we measured Smn protein levels in various tissues obtained from *Smn^2B/-^* mice. TSA administration resulted in no change in Smn protein levels in the brain and spinal cord (Fig. 5A and B). Furthermore, TSA treatment did not increase Smn protein levels in muscle, heart or liver of *Smn^2B/-^* mice (Fig. 5A and B).

**Figure 6:**
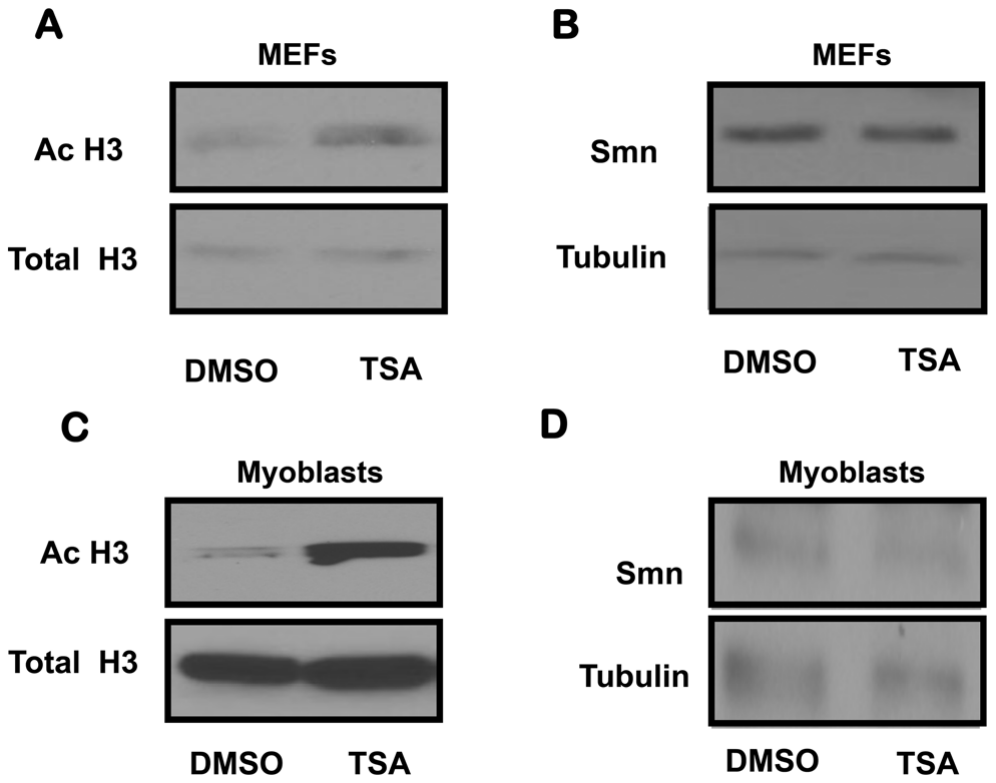
TSA treatment does not increase Smn protein levels in mouse embryonic fibroblasts and myoblasts obtained from *Smn^2B/-^* mice. Western blot analyses were performed with specific antibodies against acetylated histone H3 (**A**) or Smn (**B**) in protein extracts from TSA (100 nM) or DMSO vehicle treated MEFs obtained from *Smn^2B/-^* mice. Total histone H3 or β-tubulin serves as a loading control, respectively. (**C** **and** **D**) A similar analysis was performed in TSA (100 nM) or DMSO vehicle treated myoblasts obtained from *Smn^2B/-^* mice.

The data above suggest that TSA treatment did not increase *Smn* transcript or protein levels in various tissues obtained from *Smn^2B/-^* mice. To confirm this result, we obtained MEFs and myoblasts from *Smn^2B/-^* mice to investigate whether TSA can increase Smn protein levels *in vitro*. MEFs and myoblasts obtained from *Smn^2B/-^* mice were treated with 100 nM of TSA for 24 hours. At this dose, TSA increased the acetylation of histone H3 in MEFs and myoblasts obtained from *Smn^2B/-^* mice (Fig. 6A and C). However, TSA did not increase Smn protein levels in MEFs and myoblasts (Fig. 6B and D). We therefore conclude that *Smn* is not a specific or direct target of TSA at the time points tested, and other genes are responsible for the phenotypic and neuroprotective effects observed in *Smn^2B/-^* mice. Since HDAC inhibitors and TSA can cause global transcriptional changes, and there are no changes in *Smn* transcript or protein levels in various tissues and cells obtained from *Smn^2B/-^* mice, we suggest that the beneficial effects of TSA on the *Smn^2B/-^* phenotype are *Smn*-independent.

## Discussion

In this study, we have shown that TSA administration have beneficial effects in the *Smn^2B/-^* mouse model. Repeated administration of TSA leads to a significant improvement in survival and growth, motor behavior, motor neuron survival, NMJ size and maturity, and muscle growth. It is important to note that this was not accompanied by an increase in Smn protein or RNA levels in either tissues from TSA treated mice or in primary cell cultures treated with TSA.

The *Smn^2B^* allele is a knock-in mutation that disrupts splicing of the endogenous *Smn* gene, resulting in an increase in the production of delta7 *Smn* mRNA with a concomitant reduction of full-length *Smn* transcripts. Presenting about 15% of full-length Smn protein levels compared to the wild type allele, the *Smn^2B/-^* mice display a neurological phenotype beginning at two weeks of age [9], [10]. The data presented here suggest that the beneficial effects of TSA in the *Smn^2B/-^* mice are likely independent of changes in the endogenous *Smn* gene. Previous reports of HDAC inhibitors in mouse models of SMA have shown similar beneficial effects. Daily administration of TSA to the delta 7 mouse model from P5 to P13 lead to improved survival, attenuated weight loss and enhanced motor behavior, increased myofiber size and number, and increased anterior horn cell size [13]. Full-length *Smn* transcript levels were not increased in the brain, liver, and spinal cord of TSA treated delta 7 mice, however, there was a 1.5 fold increase in Smn protein levels in these tissues. It should be emphasized that the delta 7 model carries the human *SMN2* transgene, which has been reported to be a direct target of TSA [11]. It still remains unclear whether the small increase in SMN protein levels is sufficiently capable to cause dramatic changes in phenotype. Regardless, our results demonstrate that TSA exerts beneficial effects on a mouse model of SMA, which lacks the human *SMN2* transgene, without increasing Smn levels. The benefits of TSA are therefore likely to extend beyond simply increasing Smn levels, and are likely to be targeting additional neuroprotective pathways, which can decrease motor neuron loss and phenotypic severity. It is now important to determine how TSA is conferring these beneficial effects.

The Smn response to TSA in the delta 7 model of SMA varies significantly from tissue to tissue at both transcript and protein levels [13]. The human *SMN1* and *SMN2* promoters are virtually identical in both sequence and activity [28]. The human *SMN* and mouse *Smn* promoters share sequence homology in seven highly conserved regions that contain the consensus sequence for a number of transcription factors [29]. The human *SMN* and murine *Smn* also have a highly conserved pattern of histone H3 and H4 acetylation [11]. Importantly *Smn* transcript did not increase in target neuronal tissue following TSA administration in the delta 7 model of SMA [13]. Therefore, the current available studies do not indicate a direct nor specific detectable increase in *Smn* transcript levels in response to TSA, further supporting the hypothesis that the observed beneficial effects of TSA are independent of *Smn*.

TSA treatment enhanced motor behaviour, gene expression of choline acetyltransferase (ChAT), but not ventral horn neuron number in the delta 7 SMA model [13]. In contrast, we observed a rescue of the motor neuron loss along with motor function improvement in the TSA-treated *Smn^2B/-^* mice. While severity and time of onset differ between these two models, there is no systematic comparison of SMN/Smn protein levels, correlation of SMN/Smn protein levels to motor neuron morphology and global gene expression, and extent of perturbation in motor function. Differences in these aspects can certainly influence the overall outcome of TSA treatment. Therefore, one speculation to the discrepancy in motor neuron rescue in the two studies is that there may be a certain developmental window, after which motor neuron loss is less likely reversible. TSA has general neuroprotective properties in various other cellular conditions and animal models of neurodegenerative disease. TSA increases alpha-synuclein levels and protects against glutamate induced excitotoxicity in the cerebellum and frontal cortex as well as in cortical neurons [30]. Oxidative stress and disturbed glutamate transport has been reported in type I SMA patients [31]. TSA also increases cytoprotective HSP70 levels and concurrently suppresses P53 upregulation, consequently inhibiting the effects of this proapoptotic transcription factor [32]. Other than playing a key role in neuroprotection, HSP70 also plays a role in the muscle and protects against the atrophic features also present in SMA [33]. Aberrant apoptosis through the downregulation of Bcl-2 proteins in motor neurons of type I SMA patients is well documented [34] and TSA can increase the levels of Bcl-2 [32]. TSA also increases neurotrophic support to existing neurons by robustly increasing the transcription of BDNF and GDNF [35]. Therefore, TSA utilizes multiple neuroprotective pathways to improve neuronal function.

In summary, we have shown that TSA, a potent pan-HDAC inhibitor, significantly improves the phenotype caused by Smn deficiency in the *Smn^2B/-^* intermediate mouse model of SMA. TSA treated *Smn^2B/-^* mice were significantly better on outcome measures such as survival, growth, motor behavior, motor neuron survival, NMJ maturity, and muscle histology. These beneficial changes were not accompanied by an increase in Smn levels in various tissues and cells obtained from the *Smn^2B/-^* mice. Therefore, the beneficial effects that we observed are likely mediated through *Smn-*independent pathways. Identification of these pathways will be of therapeutic value in SMA.
